# Unraveling the disease pathogenesis behind lethal hydrolethalus syndrome revealed multiple changes in molecular and cellular level

**DOI:** 10.1186/1755-8417-2-2

**Published:** 2009-04-28

**Authors:** Heli Honkala, Jenni Lahtela, Heli Fox, Massimiliano Gentile, Niklas Pakkasjärvi, Riitta Salonen, Kirmo Wartiovaara, Matti Jauhiainen, Marjo Kestilä

**Affiliations:** 1 National Institute for Health and Welfare and FIMM, Institute for Molecular Medicine Finland, Helsinki, Finland; 2 Medical Biochemistry and Developmental Biology, Institute of Biomedicine, University of Helsinki, Helsinki, Finland; 3 Biomedicum Genomics, University of Helsinki, Helsinki, Finland; 4 Department of Medical Genetics, Väestöliitto, Helsinki, Finland

## Abstract

**Background:**

Hydrolethalus syndrome (HLS) is a severe fetal malformation syndrome characterized by multiple developmental anomalies, including central nervous system (CNS) malformation such as hydrocephaly and absent midline structures of the brain, micrognathia, defective lobation of the lungs and polydactyly. Microscopically, immature cerebral cortex, abnormalities in radial glial cells and hypothalamic hamartoma are among key findings in the CNS of HLS fetuses. HLS is caused by a substitution of aspartic acid by glycine in the HYLS1 protein, whose function was previously unknown.

**Results:**

To provide insight into the disease mechanism(s) of this lethal disorder we have studied different aspects of HLS and HYLS1. A genome-wide gene expression analysis indicated several upregulated genes in cell cycle regulatory cascades and in specific signal transduction pathways while many downregulated genes were associated with lipid metabolism. These changes were supported by findings in functional cell biology studies, which revealed an increased cell cycle rate and a decreased amount of apoptosis in HLS neuronal progenitor cells. Also, changes in lipid metabolism gene expression were reflected by a significant increase in the cholesterol levels of HLS liver tissues. In addition, based on our functional studies of HYLS1, we propose that HYLS1 is a transcriptional regulator that shuffles between the cytoplasm and the nucleus, and that when HYLS1 is mutated its function is significantly altered.

**Conclusion:**

In this study, we have shown that the *HYLS1* mutation has significant consequences in the cellular and tissue levels in HLS fetuses. Based on these results, it can be suggested that HYLS1 is part of the cellular transcriptional regulatory machinery and that the genetic defect has a widespread effect during embryonic and fetal development. These findings add a significant amount of new information to the pathogenesis of HLS and strongly suggest an essential role for HYLS1 in normal fetal development.

## Background

Hydrolethalus syndrome (HLS, MIM 236680) is an autosomally recessively inherited developmental malformation syndrome leading to stillbirth or death shortly after birth. HLS is characterized by a severe central nervous system (CNS) malformation with hydrocephaly and absent midline structures of the brain. The main neuropathological findings include a unique open-book appearance of the brain midline, the 'key-hole' defect in the base of the scull, a massive accumulation of cerebrospinal fluid, a dysplastic cortex, agenesis of the hippocampi, hypoplastic cerebellum and brain stem as well as hypothalamic hamartoma. Microscopically, disrupted and abnormal radial glial formation can be seen, as well as a severely disorganized cortex with abundant primitive neuroepithelial rosettes [1]. Other clinical features include micrognathia, polydactyly of hands and feet, and defective lobation of the lungs. In addition, the amount of amniotic fluid is increased, with case reports of up to eight liters at birth [2,3]. Nowadays, HLS can be effectively detected by ultrasound scan, usually in the end of the first trimester of the pregnancy [4]. HLS is enriched in the Finnish population with an incidence of at least 1:20,000 [3].

Our earlier studies have revealed a missense mutation in a novel gene *HYLS1* as a causative mutation for HLS [5]. This mutation is an A to G transition leading to a substitution of aspartic acid 211 to glycine (D211G) in the 299 amino acid polypeptide. *HYLS1* consists of six exons with five known splice variants, but only the last exon encodes for the protein. *HYLS1* was seen to have a wide expression pattern when human fetal cDNAs were studied and the same was observed in mouse embryonic *in situ *studies. When the cellular localization of the HYLS1 protein was studied in an overexpression cell model we detected the partially different localization of wild-type (wt) and mutant forms of the protein. While the wt form localizes mainly into the cytoplasm, the mutated form partly accumulates in the nucleus forming dot-like structures [5]. The function of the HYLS1 protein is not known and it lacks any known functional domains except a low-complexity region. In addition, the protein is not homologous with any known protein family. However, the polypeptide has orthologs among other species with a conserved polypeptide domain where the mutation site is located [5].

In this study, we have had an exceptional opportunity to investigate several cell and tissue samples from HLS cases and healthy control fetuses with different methods in order to understand the disease mechanism(s) of this grievous disorder. In addition, we have obtained important novel information about the function of the normal and mutant HYLS1 protein which has a critical role during fetal development.

## Results

### Disease pathogenesis of HLS

#### Microarray analysis

Owing the dramatic phenotype of HLS fetuses and the lack of exact information on HYLS1 function, we performed genome-wide gene expression analysis of fetal skin fibroblast cell lines (HLS *N *= 3, control *N *= 4) to obtain novel data on cellular pathways influencing the HLS pathogenesis. The quality control of the samples showed that the control and HLS samples were distinctly assigned to their own clusters. At the end, we obtained a total of 802 transcripts to evaluate by analysis of variance (ANOVA; *t*-test) statistical testing and as a result, we chose a statistically significant subgroup of these to be analyzed further.

**Figure 1:**
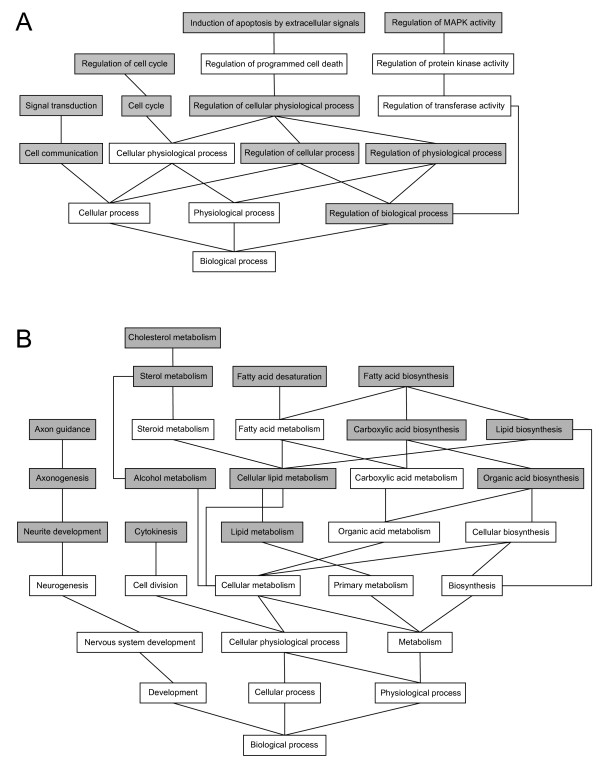
**Regulated pathways in hydrolethalus syndrome fibroblasts**. Most of the upregulated pathways (A) are involved in cell cycle regulation and signal transduction events while the downregulated pathways (B) are mostly involved in lipid metabolism. Significantly regulated pathways are marked with a gray background.

Several pathways were significantly differentially expressed between control and HLS cells (see Figure 1 and Additional file 1). Difference in the amount of expression varied from -8.02 to +13.79 in the whole data set. After examination of the transcript lists (Webgestalt program), many of the upregulated genes were associated with classification cell cycle regulation, signal transduction and the downregulated genes to the general categories of lipid metabolism and axon guidance (Figure 1). The most upregulated genes inside the classification groups included *DUSP6 *(dual specificity phosphatase 6, fold-change 7.06), *PDGFA *(platelet-derived growth factor alpha polypeptide, 3.76), *FGF5 *(fibroblast growth factor 5, 2.81), *CFLAR *(CASP8- and FADD-like apoptosis regulator, 2.63), *MAP3K5 *(mitogen-activated protein kinase kinase kinase 5, 2.92) and *CCND1 *(cyclin D1, 2.91). The downregulated genes related to lipid metabolism included *LARGE *(like-glycosyltransferase, fold-change -3.54), *SCD *(stearoyl-CoA desaturase, -3.50) and *ACAT2 *(acetyl-Coenzyme A acetyltransferase 2, -3.02). Also *LDLR *(low-density lipoprotein receptor, -2.83), *DHCR7* (7-dehydrocholesterol reductase, -2.53) and *HMGCS1 *(3-hydroxy-3-methylglutaryl-Coenzyme A synthase 1, -2.20) were downregulated in the cells derived from the HLS cases. Downregulated genes related to axon guidance included *SPON2 *(spondin 2, -7.15) and *NPR2 *(neuropilin 2, -3.19).

#### Proliferation assay and analysis of apoptosis rate

**Figure 2:**
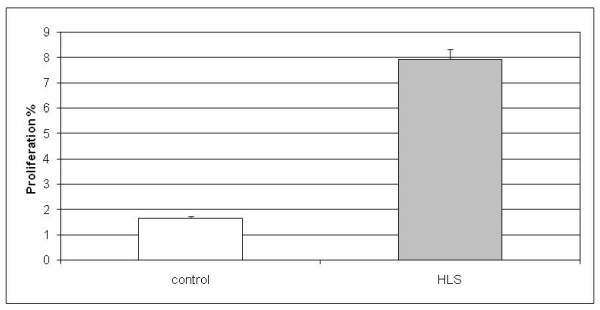
**Average percentage of bromodeoxyuridine-positive, proliferative control and hydrolethalus syndrome neuronal progenitor cells**. The amount of the proliferation is 1.7% in control cells compared with 7.9% in hydrolethalus syndrome (HLS) cells. Results are represented as the mean from three independent experiments. The *p*-value between groups is *p *< 0.001.

Since we observed upregulation of genes involved in cell cycle events in the gene expression analysis, we wanted to further study the cell cycle rate of the HLS cells compared with control cells. The proliferative activity of HLS and control neuronal progenitor cells was detected by measuring the incorporation of BrdU (bromodeoxyuridine) and the proliferating cells were identified by immunocytochemistry. The result of the BrdU assay showed significantly elevated proliferation rate of the neuronal progenitor cells obtained from the HLS fetus compared with the control cells. The amount of the proliferation was 7.9% in mutant cells, but only 1.7% in control cells, Student's *t*-test showing a statistically significant result with a *p*-value of *p *< 0.001 (Figure 2).

**Figure 3:**
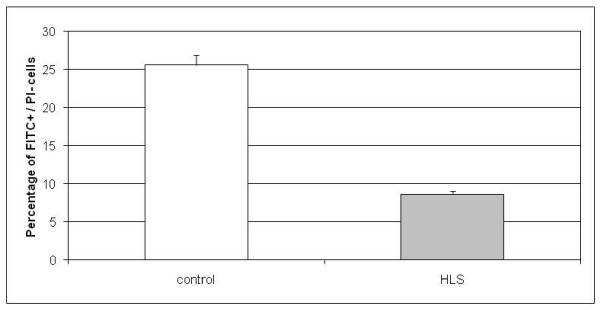
**Determination of apoptotic fractions in control and patient cell populations by Annexin V stainings**. In control cells the amount of apoptosis was 25.6% while in the hydrolethalus syndrome (HLS) cell samples apoptosis was highly reduced, being 8.7%. Results are represented as the mean from nine (control) and seven (HLS) independent experiments. The *p*-value is *p *< 0.05 between samples.

Apoptosis was measured by Annexin staining. A total of 30,000 events per sample were analyzed from which around 5,000 were actual cells. This population of real cells was analyzed in accordance with fluorescein isothiocyanate (FITC) and propidium iodide (PI) fluorescence. In control cells the amount of apoptosis was on average 25.6% while in HLS cell samples apoptosis was only 8.7% (Figure 3). These results show that apoptosis rate in HLS patient neuronal progenitor cells was highly decreased (*p *< 0.05 by Student's *t*-test). The difference in necrotic cells between control and patient cell samples was not significant (data not shown).

#### Cholesterol level measurement

**Figure 4:**
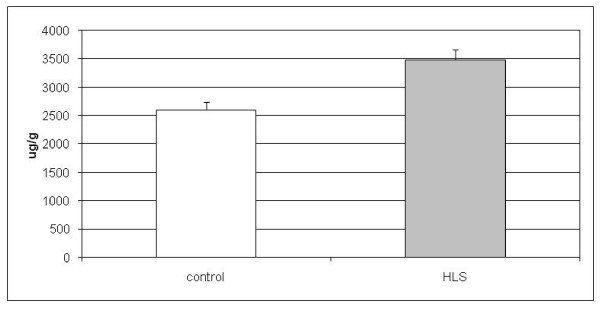
**Cholesterol levels of control and hydrolethalus syndrome liver samples**. Cholesterol level of liver (in μg cholesterol/g tissue) in healthy controls is on average 2607 μg/g and in hydrolethalus syndrome (HLS) fetuses on average 3488 μg/g, with the *p*-value being *p *< 0.01 between groups.

Since downregulation of genes involved in lipid metabolism, especially related to the cholesterol pathway, was observed in the gene expression analysis, we wanted to analyze whether the levels of lipids differed between the HLS and control samples. We had an opportunity to use liver samples collected at the autopsies from two controls and from three HLS fetuses. After the lipids were extracted from the cells, concentrations of cholesterol, phospholipids and triglycerides were determined. As a result, the average hepatic cholesterol level of HLS fetuses (3488 μg/g) was elevated 25.3% (*p *< 0.01) when compared with control samples (2607 μg/g) (Figure 4). Phospholipid as well as triglyceride levels did not differ in liver samples between the two groups (*p *> 0.05) (data not shown). This result suggests a disturbance in hepatic sterol balance in affected cells and supports the microarray findings of differential expression in lipid pathway between the controls and the HLS cases.

### Functional studies of HYLS1

#### Nuclear export study

**Figure 5:**
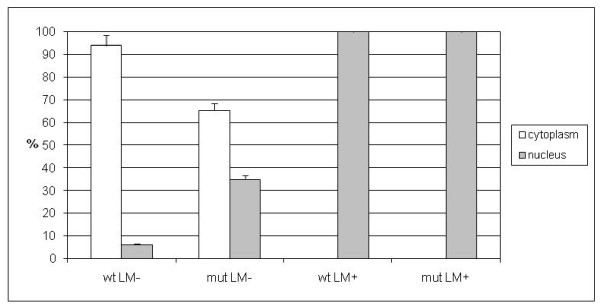
**Leptomycin B treatment of cells transfected with a construct expressing either wild-type or mutant *HYLS1***. Average percentages of cells showing either cytoplasmic (white, cells showing cytoplasmic staining only) or nuclear (gray, cells showing only nuclear or nuclear and cytoplasmic staining) localization are given. After leptomycin B treatment (LM+), both wild-type (wt) and mutant (mut) HYLS1 are showing nuclear localization that significantly differs from the untreated state (LM-) (both wt and mutant *p *< 0.001). The proportion of nuclear localization in untreated mutant cells versus wt cells is also significant (*p *< 0.001).

Leptomycin B (LMB) specifically inhibits CRM-1-mediated nuclear export, a process that has recently been shown to be important for the function of several proteins [6]. Although HYLS1 lacks the putative export sequences *in silico*, the dual subcellular localization of HYLS1 in the cytoplasm and nucleus [5] suggests that it might, in addition to most probably being transported into the nucleus, also be actively exported from it. To test this hypothesis, HEK-293 cells were transfected with a construct expressing either wt or mutant *HYLS1* and subjected to LMB. The proportion of cells exhibiting either a nuclear, cytoplasmic or combined nuclear and cytoplasmic staining pattern was measured. As a result we observed that the treatment of cells with LMB at 15 ng/ml for 6 hours led to exclusively nuclear localization of both wt and mutated HYLS1. After the treatment, both wt and mutant HYLS1 were showing nuclear localization that significantly differs from the untreated state (both wt and mutant treated state versus corresponding untreated state *p *< 0.001 by Student's *t*-test). In contrast, without LMB treatment most of the wt cells had cytoplasmic localization, but mutated cells localized both in nucleus and cytoplasm (Student's *t*-test between untreated wt and mutant *p *< 0.001) (Figure 5). Variations in the LMB concentration or treatment time had little effect on the outcome (data not shown). These results indicate that HYLS1 is shuttled between the nucleus and cytoplasm.

#### Transactivation assay

**Figure 6:**
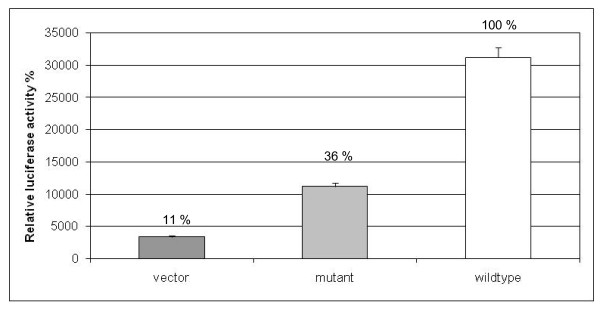
**Transcriptional activity of HYLS1**. SH-SY5Y cells were transfected with the reporter vector pG5LUC and pM1 vector alone (negative control) or with pG5LUC and wild-type or mutant HYLS1-expressing constructs. The values represent the means from three independent experiments. Here *p *< 0.01 between vector and wt HYLS1, *p *< 0.05 between vector and mutant HYLS1, *p *< 0.01 between wt HYLS1 and mutant HYLS1.

As we have hypothesized previously, HYLS1 might have a role as a transcriptional activator. To study this hypothesis we performed a transactivation assay to test whether the protein possesses transactivation capacity. For this, wt and mutant HYLS1 in fusion with the Gal4-DBD as well as the reporter vector pG5LUC were transiently expressed in the SH-SY5Y neuroblastoma cells. The wt protein was shown to activate the transcription 9.4-fold compared with a blank vector, whereas the mutant HYLS1 was capable to increase the transactivation only 3.4-fold (Figure 6). The difference between vector and wt is statistically significant (*p *< 0.01 with Student's *t*-test) as well as between vector and mutant (*p *< 0.05). Also the difference between the wt and mutant form of HYLS1 is significant with *p *< 0.01. Thus, we conclude that HYLS1 has transactivation capacity and this capacity is significantly decreased in cells containing the mutant form of the protein.

## Discussion

HLS is a severe fetal malformation syndrome with a wide spectrum of phenotypic features and with the pathogenic mutation situated in a gene with no previously known function. Therefore, we used a combination of several different research methods to unravel the disease pathogenesis behind this lethal syndrome as well as to shed light on the role of the HYLS1 protein during fetal development.

As described earlier, the structural pattern of the brain is severely disturbed in HLS fetuses both at the macroscopic and the microscopic level [1]. Frequent findings include the absence of the midline structures, a severely disorganized cortex, disrupted and abnormal formation of radial glial cells, as well as formation of primitive neuroepithelial rosettes. In current cellular studies, HLS neuronal progenitor cells showed a significantly increased proliferation rate while the amount of apoptosis was decreased. These findings are highly interesting because apoptosis is an essential and precisely controlled phenomenon of controlled cell death both in fetal development and in adulthood. When creating animal models with suppressed apoptosis rate, in many cases genetic reduction or elimination of cell death led to gross anatomical malformations or embryonic mortality, in some cases probably as a result of continued proliferation of undead cells [7]. This result nicely supports the reduced amount of apoptosis in HLS cells and further, the dramatic phenotype of the CNS and other malformations seen in HLS. Still, the relationship between the reduced rate of apoptosis and the elevated proliferation rate needs additional studies in order to explain the interaction of these findings and to understand the mechanism causing the severe malformations in the brain. It would be beneficial to clarify whether one of these phenomena is the primary and the other the secondary symptom occurring during the abnormal brain development in HLS.

Among other interesting results in microarray analysis, neuropilin 2 was found to be downregulated by over three-fold in HLS cells. Neuropilins function as receptors for some of the semaphorins, semaphorins in turn acting as chemorepulsive signal in interneuron migration during development of the brain cortex. Marin et al used a mouse model to demonstrate that interneurons expressing neuropilins avoid entering the striatum because of this repulsive signal and are thus directed to the cortex [8]. Loss of neuropilin function, in turn, increases the number of interneurons that migrate into the striatum. When the expression level of neuropilins is decreased, the correct migration pattern of interneurons could also be compromised in the brain of the HLS cases. Thus, the expression array result corresponds well with the findings of disrupted brain architecture in our previous studies [1]. Although highly speculative at the moment, the observed upregulation of *CCND1*, in turn, might in its part explain the hamartoma finding in HLS fetuses since abnormal expression levels of *CCND1 *have been linked with development of cancer [9]. Also, in a recent report, intestinal cell kinase (ICK) was found to be mutated in a syndrome sharing some overlapping symptoms with HLS and ICK was suggested to be involved in cell-cycle regulation and apoptosis during mammalian development [10]. This is of interest as kinases are crucial in several cellular functions including signal transduction and since several genes related to signal transduction and further, some genes associated with the protein kinase cascade, were upregulated in HLS neuronal progenitor cells.

When comparing HLS with other fetal malformation syndromes, Pallister-Hall syndrome (PHS, MIM 146510) [11,12] that is caused by mutations in the *GLI3* gene [13-15], probably has the most resemblance to HLS. The shared features between HLS and PHS are polydactyly, micrognathia, occasional cleft/lip palate, abnormal lobation of lungs, heart defects and hypothalamic hamartoma. GLI3 is a transcription factor of the Sonic hedgehog (SHH) pathway and *Gli3* deficient mice have been shown to have a disorganized radial glia structure in the brain cortex [16], the condition also seen in the brain of HLS cases [1]. Another syndrome resembling HLS is Smith-Lemli-Opitz syndrome (SLOS, MIM 270400) [17,18]. SLOS has a wide spectrum of phenotypic features ranging from a mild disorder with learning and behavioral problems to a lethal malformation syndrome. Prominent features of the syndrome include growth retardation, microcephaly, holoprosencephaly, ptosis, micrognathia, cleft palate and postaxial polydactyly. SLOS is caused by the mutations in the *DHCR7* gene [19,20]. These mutations lead to increased levels of 7-dehydrocholesterol and decreased cholesterol levels in patient serum [21] while interestingly in our gene expression arrays, *DHCR7* was downregulated by almost three-fold when compared with the wt cells. Also, increased cholesterol levels were observed in HLS liver samples. In addition to *DHCR7* the microarray analysis revealed other genes such as *HMGCS1*, *ACAT2 *and *LDLR *involved in the cholesterol metabolism that were downregulated. These results suggest that build-up of cholesterol in the liver causes feedback downregulation of these genes involved in the cholesterol metabolism or receptor-mediated endocytosis of cholesterol in the form of low-density lipoprotein (LDL) particles from the circulation.

Since PHS and SLOS share several similar features with HLS there is a possibility that HYLS1 is involved in similar kinds of molecular pathways as proteins behind these syndromes. Thus, it is tempting to hypothesize that HYLS1 directly or indirectly affects the function of the SHH signaling pathway. Since HYLS1 has significant transactivational capacity it could function as a transcriptional regulator in some stage of the SHH pathway as GLI3 does. On the other hand, as several genes related to lipid metabolism show downregulation in HLS cells and as SHH protein is dependent on cholesterol to function properly, HYLS1 could function indirectly in SHH signaling via cholesterol metabolism pathway instead of being directly part of the SHH pathway.

As a result of the essential new studies in cellular level, we found out that the transcriptional activity of HYLS1 is significantly decreased when mutated. In addition, we know that the subcellular localization differs between wt and mutant forms of HYLS1. Based on these results, it can be hypothesized that HYLS1 is a part of the transcription regulatory machinery and that different results between the wt and the mutant protein are a result of the conformational change in protein structure caused by the amino acid substitution. It still remains to be solved whether the amino acid change results in difficulty in the exportation of HYLS1 from the nucleus to cytoplasm, which disturbs the function of the protein. Another possibility is that the mutated HYLS1 is unable to reach the activity level required for normal function and this leads to disturbed downstream pathway regulation or that the possible conformational change in the mutated form of HYLS1 somehow disturbs normal developmental pathway(s) when it is mutated and, thus, leads to the extensive amount of severe symptoms.

## Conclusion

In this study, we have demonstrated through several analyses the significant consequences that the HYLS1mutation causes in the cellular and tissue levels. As a conclusion, we suggest that HYLS1 is a transcriptional regulator functioning in central pathway(s) related to fetal developmental processes. The results presented here add a significant amount of new information of the HLS pathogenesis and although the subject of the study is a rare malformation syndrome, these findings strongly suggest an essential role for HYLS1 in normal fetal development.

## Methods

### Subjects and samples

This study has been approved by the ethical committees of Joint Authority for the Hospital District of Helsinki and Uusimaa, Finland. The controls were age-matched healthy fetuses aborted for social reasons. The parents' consent was obtained for the collection and study of the autopsy samples.

### Cell culture

Embryonic skin fibroblast cells obtained from the control and HLS fetuses as well as commercial human embryonic kidney cell line (HEK-293) were cultured as monolayers in Dulbecco's modified Eagle's medium (DMEM) supplemented with 10% bovine calf serum, 100 units/ml penicillin and 100 units/ml streptomycin. SH-SY5Y human neuroblastoma cells were cultured as monolayers in 1:1 mixture of Ham's F12 and DMEM supplemented with 10% bovine calf serum, 100 units/ml penicillin, 100 units/ml streptomycin and 0.1% nonessential amino acids. In order to obtain neuronal progenitor cells, cortical biopsies were collected at autopsy. The autopsy was performed for each fetus within 5 hours of delivery. Neuronal progenitor cell lines were initiated by homogenizing brain tissue with pipetting and filtering prior to culture. The cells were cultured as neurospheres in serum free progenitor cell medium, consisting of DMEM supplemented with 20 ng/ml of basic fibroblast growth factor (FGF, Sigma-Aldrich, St. Louis, MO, USA), 40 ng/ml of epidermal growth factor (EGF, Sigma-Aldrich) and 10 ng/ml of leukemia inhibitory factor (LIF, Chemicon International, Temecula, CA, USA). All cells were maintained at +37°C in a 5% CO_2 _atmosphere.

### Microarray analysis

Microarray analysis was performed according to the Affymetrix standard protocol (Affymetrix, Santa Clara, California, USA). In brief, total RNA was extracted with Trizol reagent (Invitrogen, Carlsbad, California, USA) and RNeasy Mini kit (Qiagen, Venlo, the Netherlands). Quantity of the RNA was determined with UV-spectrometry and the quality was controlled with the Agilent Bioanalyzer 2100 (Agilent Technologies, Palo Alto, California, USA). The cRNA was fragmented and hybridized to Affymetrix HG-U133 Plus 2.0 chips (Affymetrix) as described in the protocol. Post-hybridization procedures including washing, staining and scanning were performed according to the Affymetrix protocol.

### Analysis of gene expression data

Cell intensity files (CEL) and chip files (CHP) were generated from images of the scanned arrays using Affymetrix Gene Chip Operating Software (Affymetrix) with the default settings recommended by the manufacturer. The CEL files are accessible through the ArrayExpress accession E-MEXP-1900 [22]. All further data processing was carried out using the GeneSpring 7.1 data analysis software (Silicon Genetics, Redwood City, California, USA). CEL files were normalized using the GC-RMA algorithm [23] with default settings, while the cross gene error model implemented in GeneSpring was used to determine cut-offs for lowest reliable signal intensities. The expression level of each gene was then equally scaled through median centering. Unreliable data was filtered out using a filtering strategy based on Affymetrix detection calls, which were extracted from the CHP files and imported into GeneSpring. In order to pass the filter genes had to score a *marginal *or *present *call in all arrays, or *absent *in all arrays pertaining to one condition while *marginal *or *present *in all arrays of the other condition. Hierarchical clustering, based on average linkage and Pearson's correlation, and principal component analysis (PCA) were performed to identify arrays with outlier behavior, compared with their biological replicates representing the same condition. A two-step process was employed to identify differentially expressed genes. First, genes with less-prominent changes in gene expression between affected subjects and controls were excluded by requiring a fold change of at least two. The remaining genes were tested for statistically significant changes in expression using Student's *t*-test for independent samples. We corrected *p*-values to account for multiple testing using the method of Benjamini and Hochberg [24], using a 12% false discovery rate as a cut-off for significance. Lists of significantly up/downregulated genes were examined for biologically relevant associations using the web-based gene set analysis toolkit WebGestalt [25,26].

### Proliferation assay

*In vitro *study of the neuronal progenitor cell proliferation was performed using the BrdU labeling method. Neuronal precursor cells (HLS *N *= 1, control *N *= 2) maintained in culture were supplied with 1:1,000 BrdU (Amersham Biosciences UK Limited, Buckinghamshire, England) and kept in nondifferentiating media for 12–15 hours. After incubation the cells were attached to coverslips with a cytospin centrifuge and fixed with 4% PFA for 20 minutes. The BrdU positive cells were stained using the BrdU antibody kit (RPN 202, Amersham Biosciences). The ratio between the whole cell content and the amount of proliferating cells was counted. The experiment was repeated three times and all samples were prepared in duplicate. Samples were grown in several aliquots and pooled together for the analysis to minimize the effects of passage and aliquot differences. Stained cells were analyzed with a Zeiss Axioplan 2 fluorescence microscope.

### Analysis of apoptosis rate

The amount of apoptosis was measured by Annexin-V-Fluos staining kit (Roche, Basel, Switzerland). Undifferentiated neurospheres, each sample (HLS *N *= 1, control *N *= 2) containing approximately 1 × 10^6 ^cells were harvested, dissociated into single cells and double stained with FITC-conjugated annexin (FA) and propidiumiodide (PI) for 15 minutes at room temperature and diluted (1:50 both) into HEPES buffer according to the manufacturer's protocol. Negative controls were treated by the same way but without one of the appropriate stainings (only FA or PI). Cells exposed to UV-light, dexamethasone or hydroxyurea were used as positive controls to ensure functionality of the assay. Unspecific labeling of FITC was excluded by treating the cells with IgG-conjugated FITC. Annexin labels both apoptotic and necrotic cells while living cells remain unstained. Necrotic cells were excluded by PI staining, which stains DNA of leaky necrotic cells only. The amount of labeled cells was measured by flow cytometry (FACSaria, BD Biosciences, San Jose, California, USA). Measurements were repeated altogether nine times for control cells and seven times for HLS cells.

### Cholesterol and phospolipid level measurement

Cholesterol and phospholipid levels were measured from fetal liver tissue from two healthy controls and two HLS fetuses. Total lipids were extracted from whole liver homogenates using the Folch extraction [27]. Briefly, frozen liver tissue was cut into 5–15 mg pieces and homogenized in a 2:1 methanol-chloroform solvent. Homogenization was performed with Precellys 24 homogenizer (Bertin technologies, Montigny-le-Bretonneux, France) according to the manufacturer's protocol with slight modifications. The homogenized media were centrifuged briefly to separate out the cell debris. The supernatant with extracted lipids was transferred to methylene chloride-purified Kimax tubes and the solvent was evaporated in a nitrogen atmosphere. The dried lipids were kept at -20°C until lipid assays. The samples were re-dissolved in methanol and cholesterol, phospholipids and triglycerides were analyzed in triplicates according to the manufacturer's protocol (Roche/Hitachi, Basel, Switzerland for cholesterol and triglycerides, Wako, Neuss, Germany for phospholipids).

### Nuclear export study

In nuclear export studies, HEK-293 cells were transfected in the following manner: 1 × 10^6 ^cells were grown on coverslips in six-well plates and transfected with 1 μg of the appropriate expression vector, containing either wt or mut *HYLS1* with the Myc sequence immediately before the start codon. LMB (Tocris Bioscience, Bristol, UK) was added to cells 24 hour post-transfection initially at 5, 10, 15 or 20 ng/ml, the cells were grown for 3, 6 or 24 hours and stained as described below. In subsequent experiments, 15 ng/ml LMB was used. Transfections of each individual vector were performed in duplicate and the study was repeated three times. For immunofluorescence studies, cells were fixed with 4% paraformaldehyde (PFA) in phosphate buffered saline (PBS; pH 7.3) at room temperature (RT) for 10 minutes and blocked and permeabilized with 0.2% saponin/0.5% bovine serum albumin (BSA) in PBS. Cells were then incubated with primary antibody (mouse anti-c-myc, Santa Cruz Biotechnology, Santa Cruz, California, USA) for 1 hour at RT. After washing, cells were incubated with anti-mouse Texas Red secondary antibody for 40 minutes at RT. After final washes, cells were mounted onto microscope slides with Vectashield hard set mounting medium containing 4',6-diamidino-2-phenylindole (DAPI; Vector Laboratories) and the data was acquired using an Axioplan Z imaging fluorescence microscope and AxioVision Rel. 4.6 program. The proportion of cells showing either cytoplasmic, nuclear or combined cytoplasmic and nuclear staining was calculated.

### Transactivation assay

SH-SY5Y cells were seeded onto 12-well plates at 1.2 × 10^5 ^cells per well. The cells were transfected 24 hours after seeding with 750 ng of wt or mutant human *HYLS1* cloned in fusion with Gal4-DBD. In all transfections, 200 ng of reporter vector pG5-LUC (Promega, Mannheim, Germany) containing Gal4 binding sites was used. The pM1 vector was used as a negative control. The transfections were performed using the Fugene HD transfection reagent (Roche) according the manufacturer's instructions. The cells were harvested 48 hours after transfection and the luciferase activity was determined using the Luciferase Assay System (Promega). The luciferase activities were normalized according to the total protein concentration of the lysates which were determined by biuret reaction (Novagen, Madison, Wisconsin, USA). All transfections were performed in triplicate and repeated three times.

## Competing interests

The authors declare that they have no competing interests.

## Authors' contributions

HH designed this study, participated in all experiments and provided the proliferation assay, nuclear export study and lipid measurements, provided the final analyses of the functional studies, participated in sample collection and drafted the manuscript. JL provided functional studies related to the transactivation assay and assisted HH in other experiments. HF provided the analysis of the apoptosis rate using neuronal progenitor cells. MG participated in microarray data analysis. NP participated in the proliferation assay of the neuronal progenitor cells and in control sample collection. RS performed the HLS sample collection. KW participated in design and supervision of the neuronal progenitor cell related studies. MJ designed and supervised the lipid studies. MK designed and supervised this study, obtained funding and helped to draft the manuscript. All authors contributed to the revision of the drafted manuscript, and read and approved the final manuscript.

## Supplementary Material

Additional file 1 **Table S1**. Differentially expressed genes in HLS. Click here for file
