# Supplementary material for: Unraveling the disease pathogenesis behind lethal hydrolethalus syndrome revealed multiple changes in molecular and cellular level
Source: Pathogenetics. 2009 Apr 28;2:2. doi: 10.1186/1755-8417-2-2 (PMC2686686; doi:10.1186/1755-8417-2-2)
Supplement: Additional file 1 — Table S1. Differentially expressed genes in HLS. [file 1755-8417-2-2-S1.doc]

**Supplementary Table 1. Differentially expressed genes in HLS.**

| **Upregulated in affecteds** | |  | **Downregulated in affecteds** | |
| --- | --- | --- | --- | --- |
| **Name** | **Fold change** |  | **Name** | **Fold Change** |
| PRG1 | 13.79 |  | FGFR2 | -8.02 |
| KRTAP1-5 | 10.46 |  | SPON2 | -7.15 |
| PTGS2 | 7.12 |  | EPSTI1 | -5.80 |
| DUSP6 | 7.06 |  | FLJ10312 | -5.48 |
| RAB3B | 5.18 |  | BACH2 | -5.07 |
| LOC285705 | 4.17 |  | C10orf58 | -4.63 |
| ARRDC4 | 4.00 |  | CYYR1 | -3.85 |
| PDGFA | 3.76 |  | PEG10 | -3.69 |
| RORA | 3.65 |  | LARGE | -3.54 |
| SPOCK | 3.46 |  | KIAA1912 | -3.51 |
| WNT5A | 3.15 |  | SCD | -3.50 |
| AK3 | 3.10 |  | C1QTNF5 | -3.48 |
| TCF8 | 3.04 |  | INSIG1 | -3.25 |
| ARHGAP22 | 2.92 |  | NRP2 | -3.19 |
| MAP3K5 | 2.92 |  | ACAT2 | -3.02 |
| PGK1 | 2.92 |  | MX1 | -2.85 |
| CCND1 | 2.91 |  | LDLR | -2.83 |
| STARD13 | 2.82 |  | TMSNB | -2.72 |
| FGF5 | 2.81 |  | ZNF503 | -2.69 |
| RAPH1 | 2.77 |  | GBP1 | -2.62 |
| TXNIP | 2.76 |  | MYH10 | -2.60 |
| SPRY2 | 2.75 |  | FADS2 | -2.53 |
| CFLAR | 2.63 |  | DHCR7 | -2.53 |
| PRKX | 2.61 |  | FABP5 | -2.46 |
| EGFL3 | 2.54 |  | SNX8 | -2.40 |
| GLRB | 2.53 |  | MLP | -2.37 |
| FN1 | 2.42 |  | FKBP5 | -2.36 |
| TIPARP | 2.42 |  | HMGB3 | -2.35 |
| SERPINE1 | 2.40 |  | WDR18 | -2.34 |
| C10orf45 | 2.31 |  | SEPT6 | -2.32 |
| ETV5 | 2.30 |  | C9orf19 | -2.29 |
| ADAMTS1 | 2.29 |  | HMGCS1 | -2.20 |
| FLJ14011 | 2.25 |  | DOCK1 | -2.17 |
| THBS1 | 2.19 |  | EPHA3 | -2.13 |
| HPCAL1 | 2.16 |  | DKFZp761P0423 | -2.12 |
| LOC284267 | 2.04 |  | PAFAH1B3 | -2.11 |
| FLJ33215 | 2.04 |  | SFRS1 | -2.06 |
| FLJ10618 | 2.03 |  | MN1 | -2.04 |
|  |  |  | GPC4 | -2.02 |
